# Supplementary material for: The Use of Micro-Ribbons and Micro-Fibres in the Formulation of 3D Printed Fast Dissolving Oral Films
Source: Pharmaceuticals (Basel). 2023 Jan 5;16(1):79. doi: 10.3390/ph16010079 (PMC9862838; doi:10.3390/ph16010079)
Supplement: Supplementary file 1 [file pharmaceuticals-16-00079-s001.zip › pharmaceuticals-2052961-supplementary.pdf]

Table S1. The weight percentage compositions of various ingredients in formulations of FDFs. Apart from FS1 and FS2, these formulations were not printable, although filaments were formed.

Formulations	PVP 40K	PVP 10K	PEO 100K	PEO 200K	PVA	PCM	Croscar mellose	Chi-MR	C500	C1000	C2000	Chi
**FS1**	-	-	-	-	100	-	-	-	-	-	-	-
**FS2**	-	-	-	-	70	30	-	-	-	-	-	-
**FS3**	-	-	-	-	61	30	7	2	-	-	-	-
**FS4**	-	-	-	-	60	30	7	3				-
**FS5**	-	-	-	-	67	30		3				-
**FS6**	100				-	-	-	-	-	-	-	-
**FS7**	33	-		30		30	7					
**FS8**	33	-	29.5			30	7	0.5				
**FS9**	33	-	29			30	7	1				
**FS10**	33	-	28			30	7	2				
**FS11**	33	-	27			30	7	3				
**FS12**	33	-	25			30	7	5				
**FS13**	33	-	20			30	7		10			
**FS14**	40	-				30			30			
**FS15**		100										
**FS16**		33	30			30	7					
**FS17**		33	25			30	7			5		

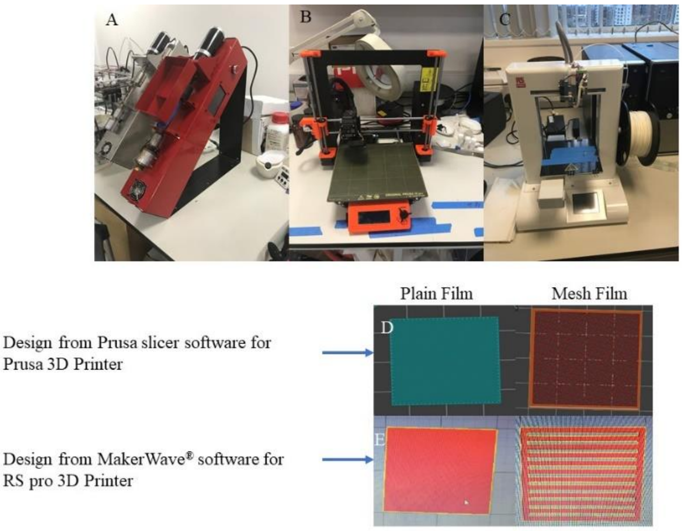

Figure S1. Photos representing A) the Noztek extruder, B) FDM Prusa® i3 MK3S 3D printer, C) RS PRO® IdeaWerk 3D printer. CAD images of the designs of plain and

mesh films by D) the Prusa slicer software, E) the MakerWave® software used by the RS-Pro 3D printer.

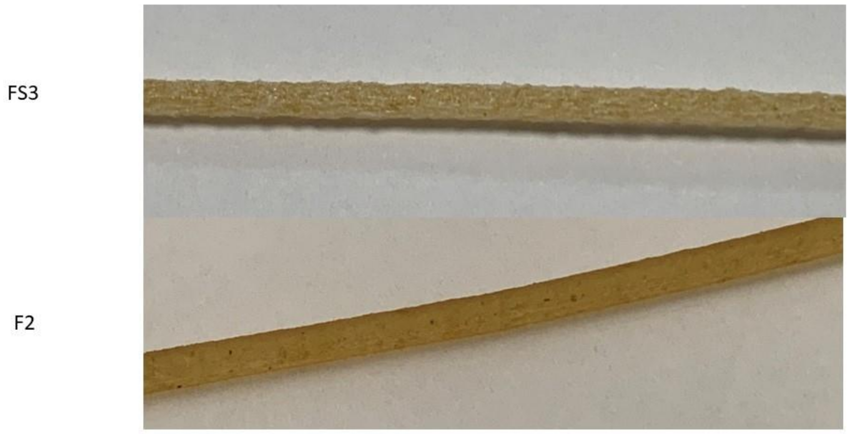

Figure S2. representing the rough surface of filaments from formulations FS3, and smooth surface of filaments from formulation F2.

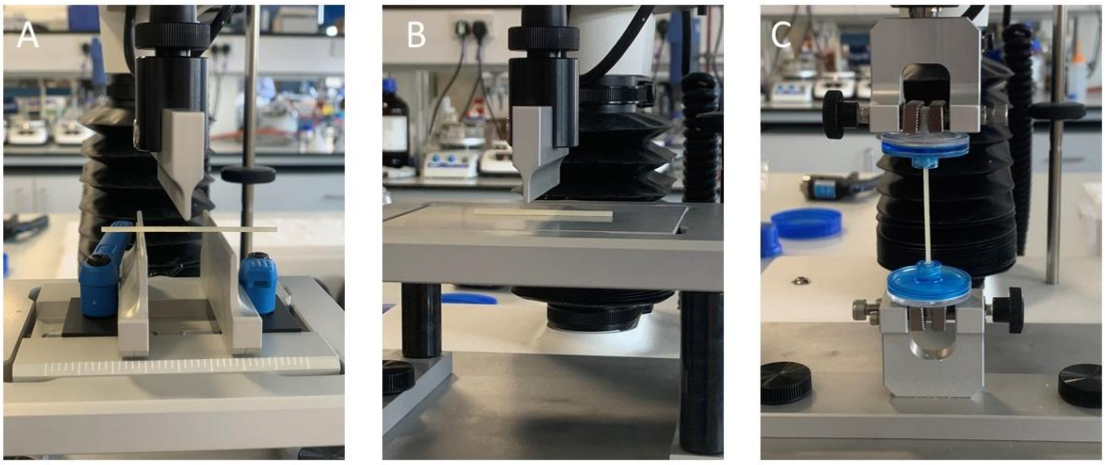

Figure S3. Evaluating the mechanical properties of the filaments using a, A) 3-point bend probe set, B) stiffness probe set, C) resistance probe set.

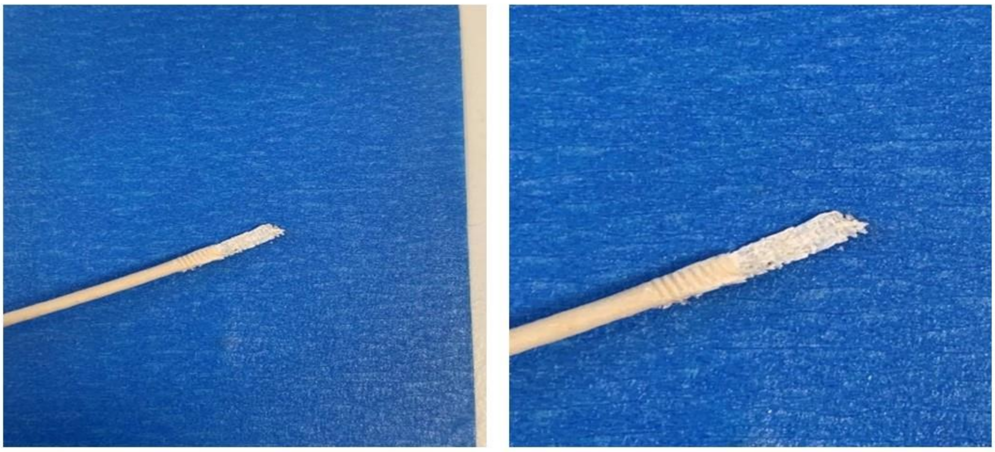

Figure S4. Representing a crushed and squeezed filament of formulations FS6 in the gear mechanism of the 3D printers.

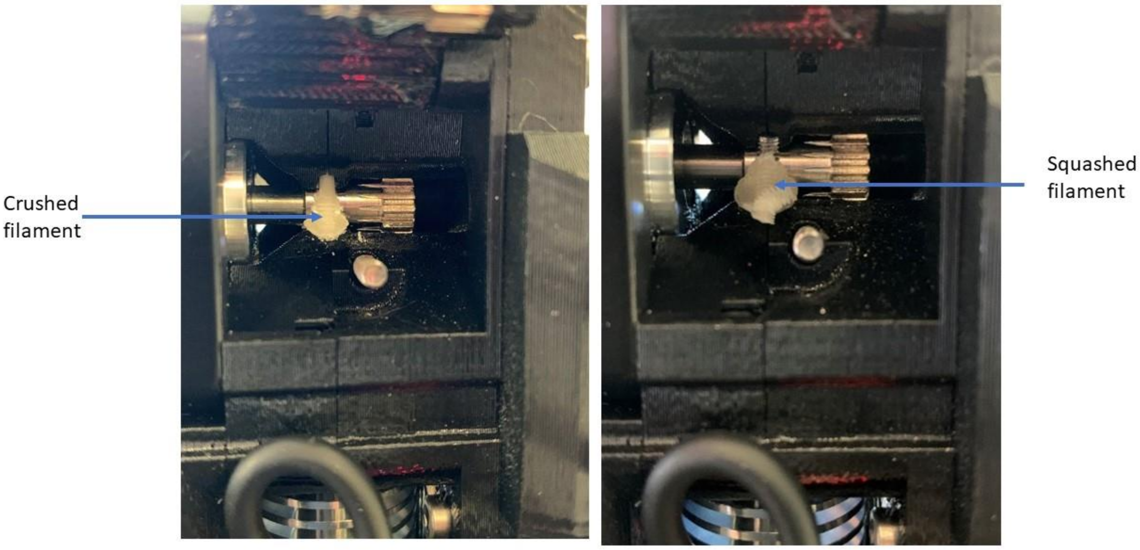

Figure S5. Demonstrating squashed/crushed filaments in the printer head of the Prusa 3D printer.

A

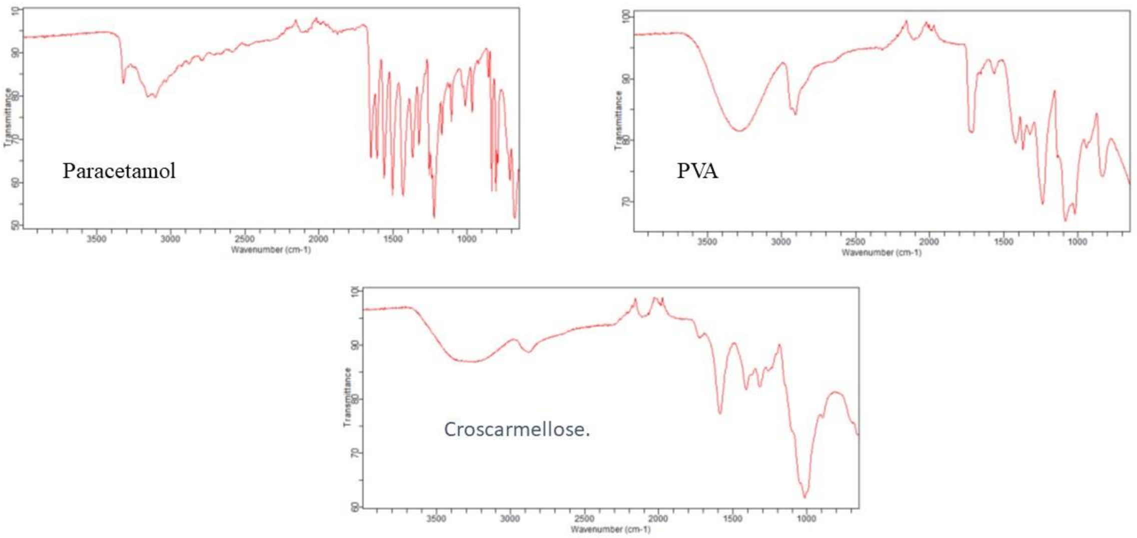

Figure S6. FTIR spectra of A) Paracetamol, B) PVA, C) Croscarmellose.
